# Low-cost customized cranioplasty using a 3D digital printing model: a case report

**DOI:** 10.1186/s41205-018-0026-7

**Published:** 2018-04-12

**Authors:** Abel De La Peña, Javier De La Peña-Brambila, Juan Pérez-De La Torre, Miguel Ochoa, Guillermo J. Gallardo

**Affiliations:** 1 Plastic and Reconstructive Surgeon, Plastic Surgery Institute, Mexico City, Mexico; 2 Maxillofacial Surgeon, PhD, Hospital Civil de Guadalajara “Fray Antonio Alcalde”, Guadalajara, Jalisco Mexico; 3 Plastic and Reconstructive Surgeon, Hospital Civil de Guadalajara “Fray Antonio Alcalde”, Guadalajara, Jalisco Mexico; 4 Neurosurgeon, Hospital Civil de Guadalajara “Fray Antonio Alcalde”, Guadalajara, Jalisco Mexico; 5 Plastic and Reconstructive Surgeon, Plastic Surgery Institute, Mexico City, Mexico

**Keywords:** Cranioplasty, Cranial implant, Skull defects, Polymethylmethacrylate resin, PMMA prosthesis, Cranial vault reconstruction, Low cost prosthesis 3D printing

## Abstract

**Background:**

Cranial defects usually occur after trauma, neurosurgical procedures like decompressive craniotomy, tumour resections, infection and congenital defects. The purpose of cranial vault repair is to protect the underlying brain tissue, to reduce any localized pain and patient anxiety, and improve cranial aesthetics. Cranioplasty is a frequent neurosurgical procedure achieved with the aid of cranial prosthesis made from materials such as: titanium, autologous bone, ceramics and polymers. Prosthesis production is often costly and requires complex intraoperative processes. Implant customized manufacturing for craniopathies allows for a precise and anatomical reconstruction in a shorter operating time compared to other conventional techniques. We present a simple, low-cost method for prosthesis manufacturing that ensures surgical success.

**Case presentation:**

Two patients with cranial defects are presented to describe the three-dimensional (3D) printing technique for cranial reconstruction. A digital prosthesis model is designed and manufactured with the aid of a 3D computed tomography. Both the data of large sized cranial defects and the prosthesis are transferred to a 3D printer to obtain a physical model in poly-lactic acid which is then used in a laboratory to cast the final customised prosthesis in polymethyl methacrylate (PMMA).

**Conclusions:**

A precise compliance of the prosthesis to the osseous defect was achieved. At the 6 month postoperative follow-up no complications were observed i.e. rejection, toxicity, local or systemic infection, and the aesthetic change was very significant and satisfactory. Customized 3D PMMA prosthesis offers cost advantages, a great aesthetic result, reduced operating time and good biocompatibility.

## Background

Loss of a body part has significant repercussions for any individual. The absence of a body part has a great influence in a person’s physical state and state of mind, and causes social interaction difficulty; which frequently limits their hope of recovery [1]. Lack of continuity of the cranial vault bones is usually secondary to severe head injury, but can also be secondary to neurosurgical procedures e.g. decompressive craniotomy or tumor resection. Infections, especially osteomyelitis and congenital anomalies or iatrogenic disorders can cause these type of defects [2].

In certain cases, cranial defects can become quite extensive or involve adjacent tissue damage. On the one hand, atmospheric pressure on the defect has a direct effect on intracranial structures causing symptoms like headache, confusion, irritability, psychiatric symptoms, contralateral weight sensation and, epilepsy [3]. On the other hand, osseous cranial defects cause aesthetic abnormalities such as herniation or depressions that may severely affect the patient’s quality of life [4].

Repair of the cranial defects has as a main goal to protect the underlying brain tissue, to decrease pain at the site of the defect, improve the appearance and decrease the patient’s anxiety [5]. The human body cannot regenerate a lost body part, but reconstruction can be obtained through a multi-disciplinary approach and the placement of a prosthesis. Cranioplasty is one of the oldest neurosurgical procedures, practiced since 3000 BC [6].

For centuries, several materials have been tested to cover osseous defects including coconut shells, allogenic and xenogenic bone grafts, metals and more recently, biosynthetic materials such as resins and ceramics [7]. Methyl methacrylate was used for the first time in 1941; since then, many other derivatives have been used in the operating room [8]. The tendency with any alloplastic material is to increase its biocompatibility. Cranioplasties done with alloplastic materials are already a well-accepted treatment method. The ideal characteristics of prosthetic materials are their inability to cause inflammatory reactions, non-allergenicity or inability to cause hypersensitivity, chemical inertness, non-carcinogenicity, ability to withstand strain and tension, capacity to be sterilized and to be molded into the desired shape when fabricated [9]. Titanium prefabricated prosthesis have some disadvantages, for example, thermal conduction, little chance of intraoperative modifications and a high cost [10]. Intraoperatively produced polymethyl methacrylate (PMMA) prostheses require complex procedures such as: preparation of the mixture with direct contact with the dura mater that can produce exothermal reactions or produce toxic monomers during surgery, implant adjustments for osseous adaptation that causes an increase in operating time. Intraoperative implant adjustments may cause poor aesthetic results in large and complex defects [11]. The advantages of PMMA are the following: it is low cost, no donor is required, it is lightweight, strong, inert, radiolucent, non-ferromagnetic and stable. The disadvantages are that it has a low adherence to the surrounding tissue, it may cause tissue reactions (subcutaneous seroma), and may be bulky in some areas like the orbital rim [12].

Computer assisted design and prosthetic material modeling result in an excellent cosmetic outcome, and reduce operating time necessary for implant placement [13].

## Case presentation

### Case report No. 1

**Fig. 1:**
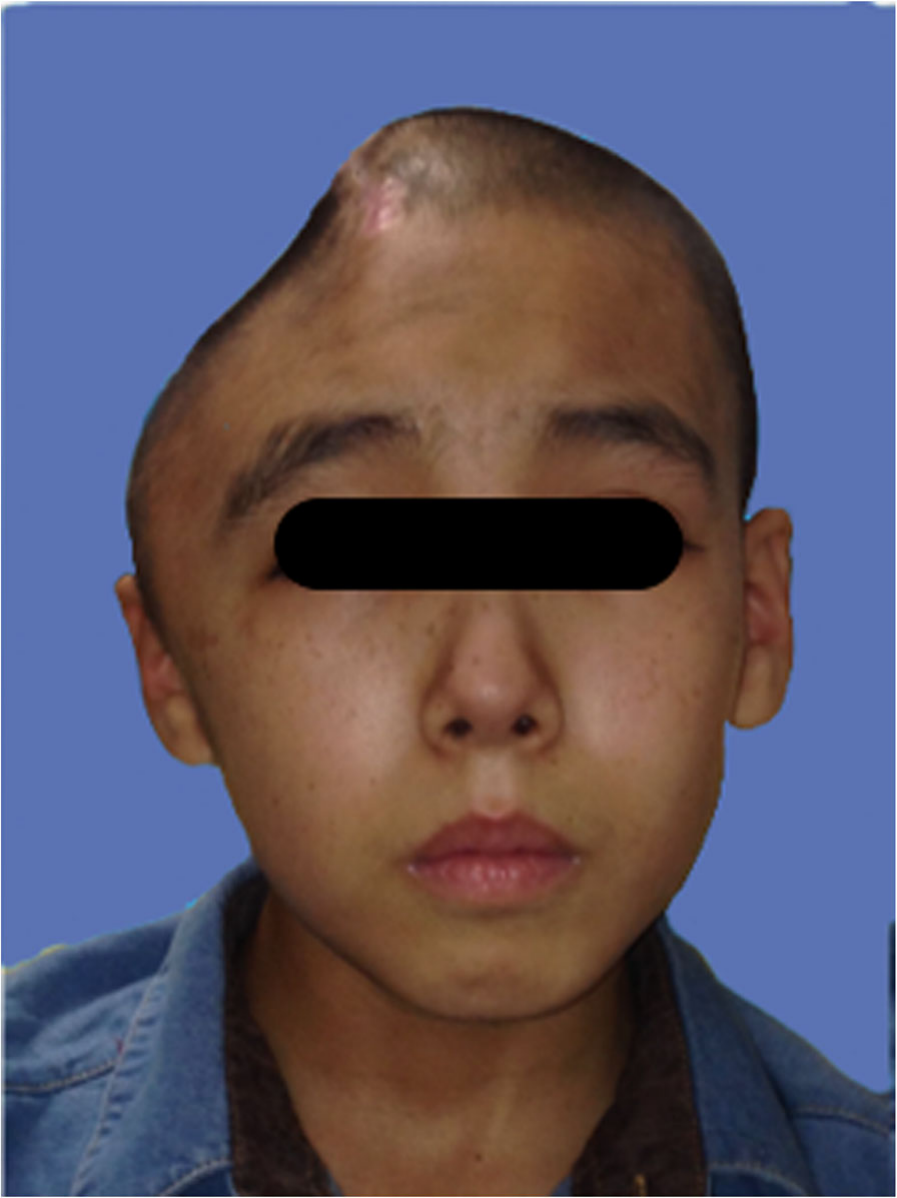
Frontal view of the cranial defect after craniotomy

**Fig. 2:**
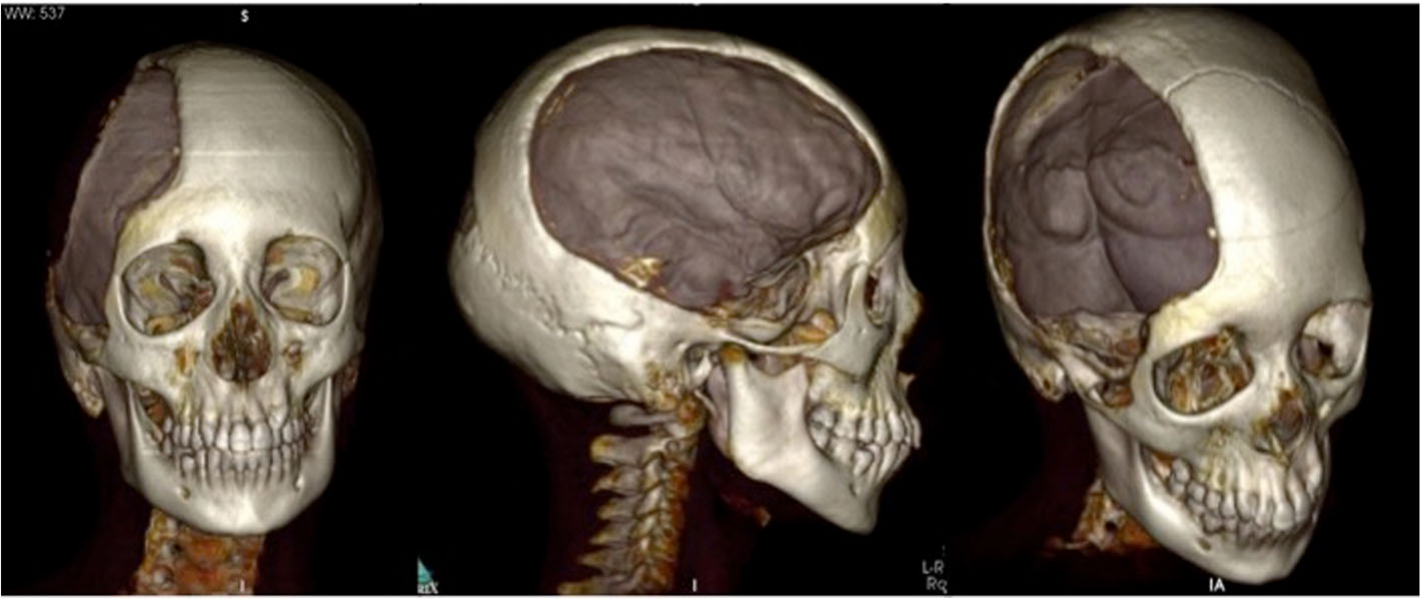
CT showing a 12 × 12 cm right cranial defect. The prosthetic design was done using a computer system (DICOM) to design and produce a customized prosthesis in acrylic resin

**Fig. 3:**
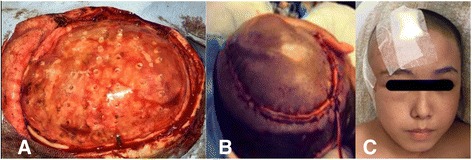
**A** Prosthesis placement on the patient. **B** Immediate postoperative period. **C** Patient before being discharged (2 days after surgery)

A 10 year old male patient presented with a severe head trauma after falling from a roof that required an immediate decompressive craniotomy (Fig. 1). The patient was hospitalized in the ICU for 8 days, after which the cerebral oedema resolved, and he was then discharged. Three months later, he was referred to the Plastic Surgery Department by the Department of Neurosurgery to plan the patient’s treatment and his cranial reconstruction. He was assessed by our department and was found to be alert and oriented, calm, and cooperative. A CT revealed a 12 cm by 12 cm defect (Fig. 2). The prosthesis was fashioned and the patient was operated 10 days later (Fig. 3). The thickness of the implant produced for this case was of 5 mm. The thickness of the prosthesis matches the patient’s cranial vault thickness. The patient was discharged from the hospital 2 days after surgery, he immediately started a physical therapy program and he returned to school after 2 months of therapy without sequelae or neurologic damage. The patient said that his headache subsided, since the prosthesis relieves atmospheric pressure on brain tissue.

### Case report No. 2

**Fig. 4:**
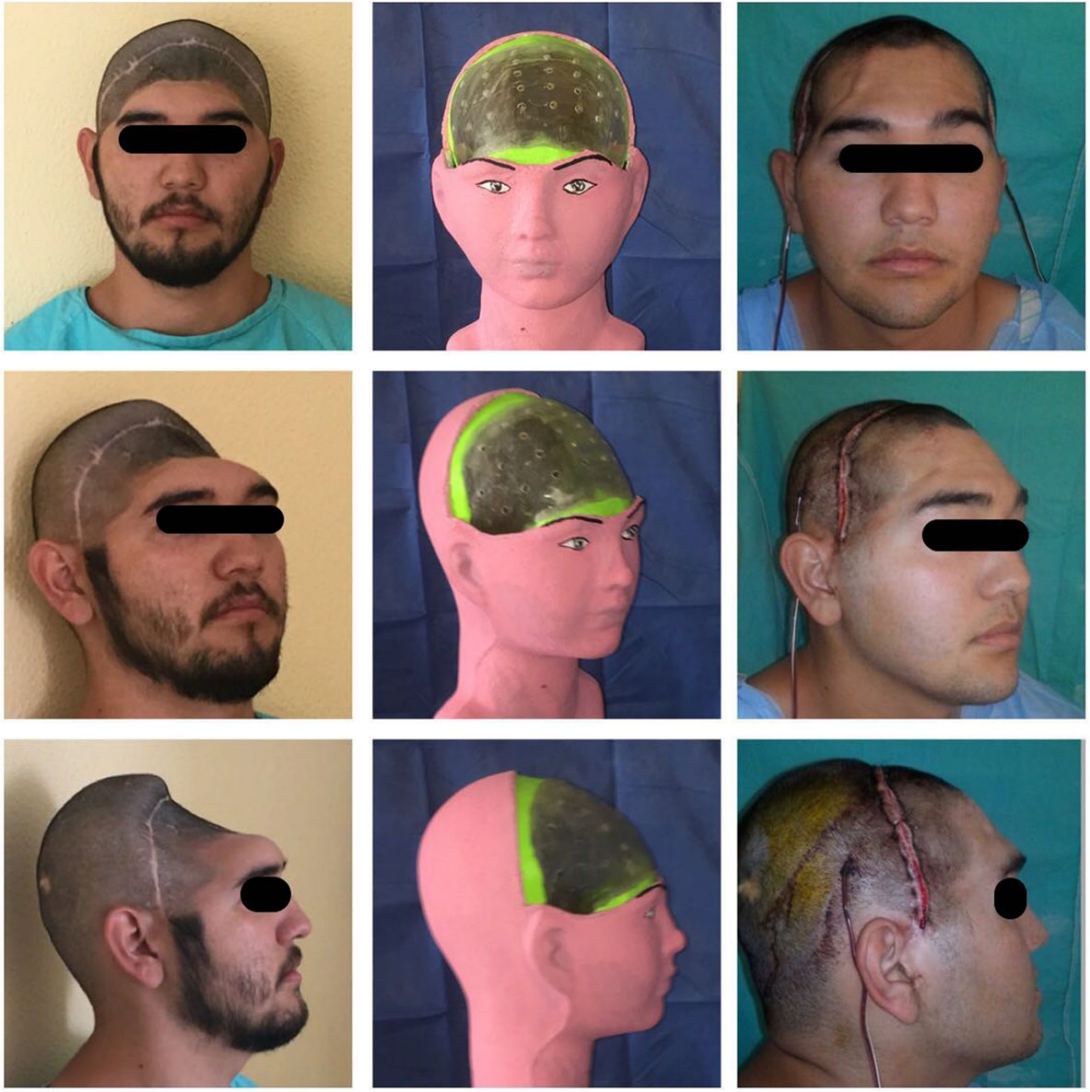
Preoperative and immediate postoperative photos. Left column, preoperative patient status. Center column, PLA prosthesis adjusted to the cranial defect on a mannequin. Right column, immediate postoperative patient status

A 17 year old male patient presented after a motorcycle accident where he was not wearing a helmet. Upon his arrival at the hospital with a diagnosis of TBI (traumatic brain injury), he was evaluated by the Department of Neurosurgery who decided to treat the patient with a decompressive craniotomy. A CT was done to complete the diagnosis and plan the surgery. The patient had a bifrontal 20 cm cranial defect from the temporal fossa of one side to the other and 10 cm vertical distance (Fig. 4). The thickness of the implant in this case was of 6 mm. It is 1 mm thicker than that of the previous case due to patient anatomic differences in diploe thickness. After surgery, he remained hospitalized for 2 weeks before discharging him. He was referred to us after 3 months of physical therapy once the Neurosurgical Department considered cranial reconstruction feasible. The patient was evaluated and was found to have no neurologic damage and an intact scalp. The patient was deemed an excellent candidate for the placement of a 3D PMMA pre-operative manufactured cranial prosthesis, 2 days after which the patient had a good evolution and was discharged. After 2 months, the patient returned to his normal school activities.

In Mexico these procedures have to be payed in full by the patient, limiting their reach and practice in the general population since they are costly. A customized titanium implant costs around US$5000, and those made from PEEK around US$7000 or more depending on their size. The customized prostheses proposed by the authors have a cost of about US$600, including the digital design, printing of a 3D prototype and the PMMA prosthesis itself. Both titanium and PMMA are the most commonly used alloplastic materials [14, 15].

### Prosthesis digital design

The CT scan data is stored in the standard format DICOM (Digital Images and Communications in Medicine) which allows generating an interface between the medical equipment and any other device to visualize the images. Through the DICOM viewer, Osirix® generated a three-dimensional reconstruction of all the CT cross-sectional images. A bone filter is applied in order to only observe the bone structure, achieved by taking as reference its attenuation degree.

The implant is generated using the software of the computer-aided design (CAD) ZBrush 4R5® since Osirix® is only a viewer.

**Fig. 5:**
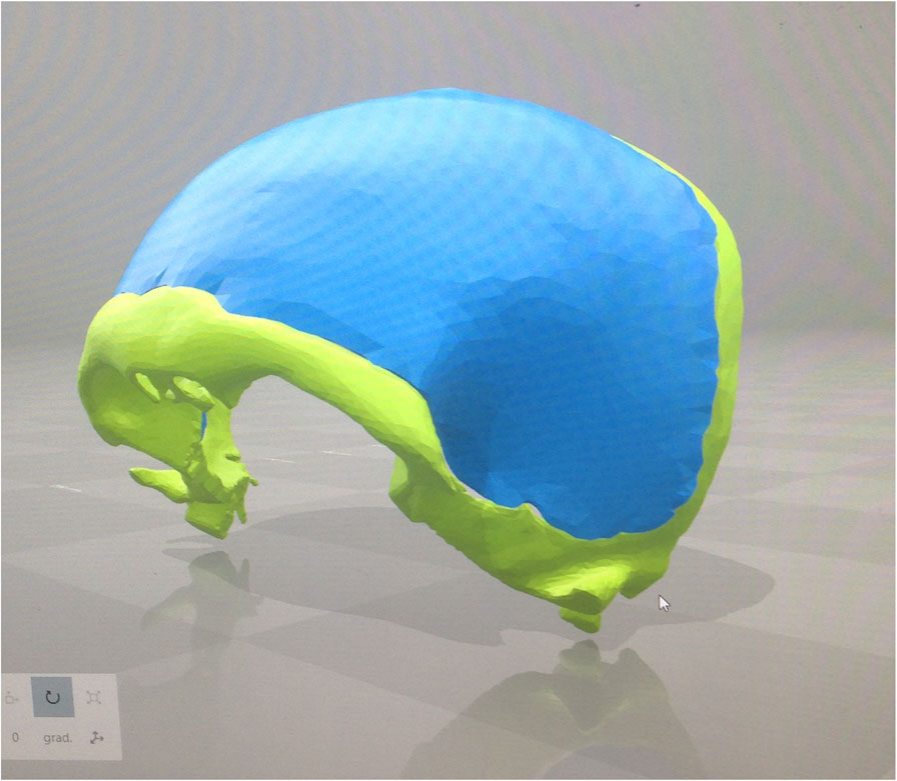
Digital design of the customized prosthesis

The implant piece, Vimplant, is generated from the CT skeletal reconstruction of the patient’s cranium. With volumetric reconstruction, Vtotal, and considering its symmetry; an imaginary division is made on the sagittal plane, obtaining two volumes, Vleft and Vright. A boolean operation is applied to the volumes Vleft and Vright: Vimplant = Vleft - Vright (Fig. 5).

The implant design must have a precise shape and volume according to each patient’s cranial anatomy. Finally, the data is exported in a stereolithography extension file (STL) and fed to the printer.

### 3D printing

**Fig. 6:**
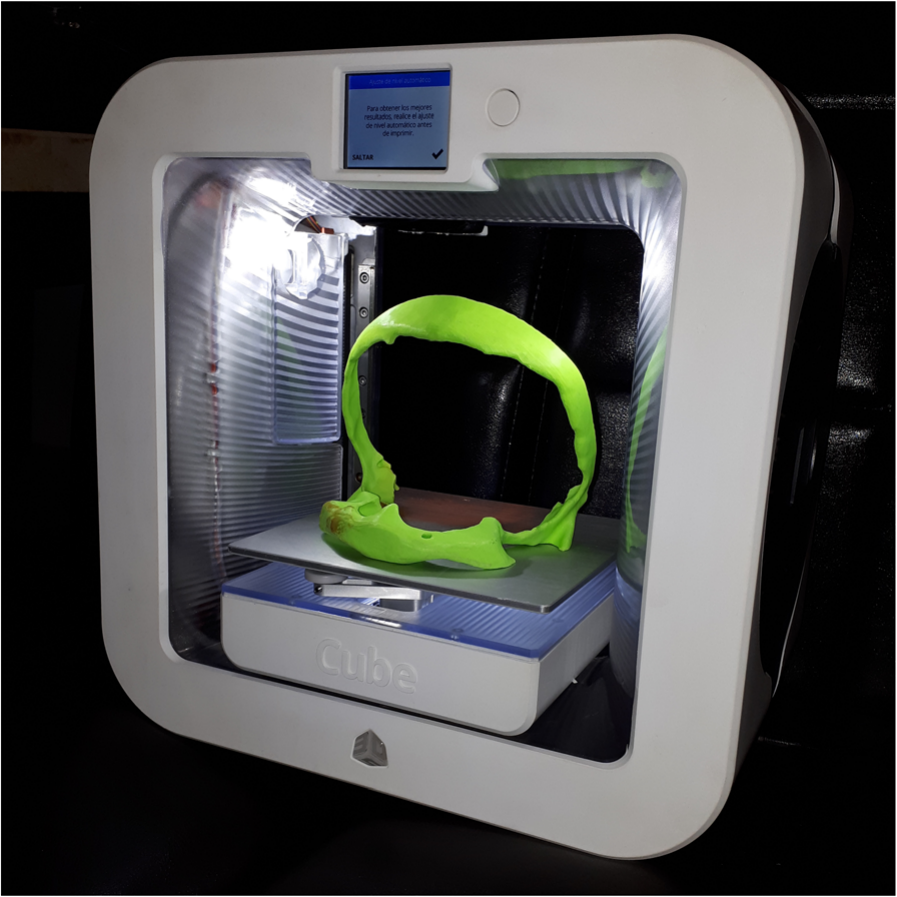
3D printing of the cranial defect and of the customized PLA prosthesis

**Fig. 7:**
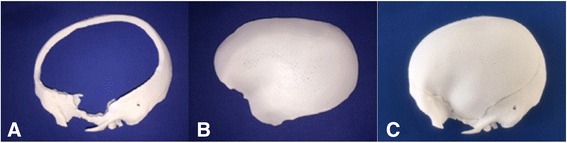
**A** Cranial defect printout. **B** PLA customized prosthesis. **C** Assembled models to test precision

A CUBE 3D (from 3D System) printer is used to print out a PLA prosthesis from the STL file using a fused deposition model by means of a 1,75 mm filament at a 260 °C extruder temperature (Fig. 6). Once the printing process is finished, which takes about 20 h, the scaffolds are removed with a low-speed motor and a carbide bur and the adjustment is verified (Fig. 7). The prosthesis is then taken to the dental laboratory.

### Dental laboratory

**Fig. 8:**
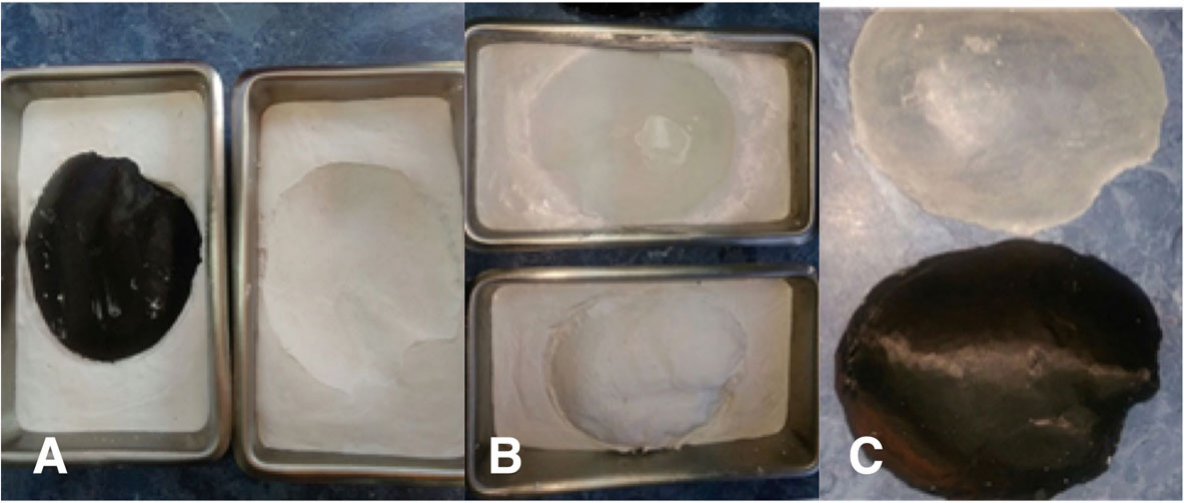
**A** PLA prosthesis plaster impressions in metal containers. **B** Internal and external mold filling with PMMA. **C**. Comparison of the PMMA prosthesis to be implanted as an identical replica of the PLA prosthesis

**Fig. 9:**
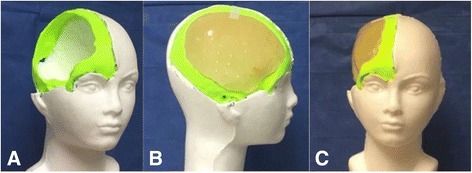
**A** Oblique mannequin view showing the 3D printout of the rim surrounding the defect. **B** Lateral view of the customized implant placed on the rim to cover the defect. **C** Frontal view of prosthesis to cranium coaptation

**Fig. 10:**
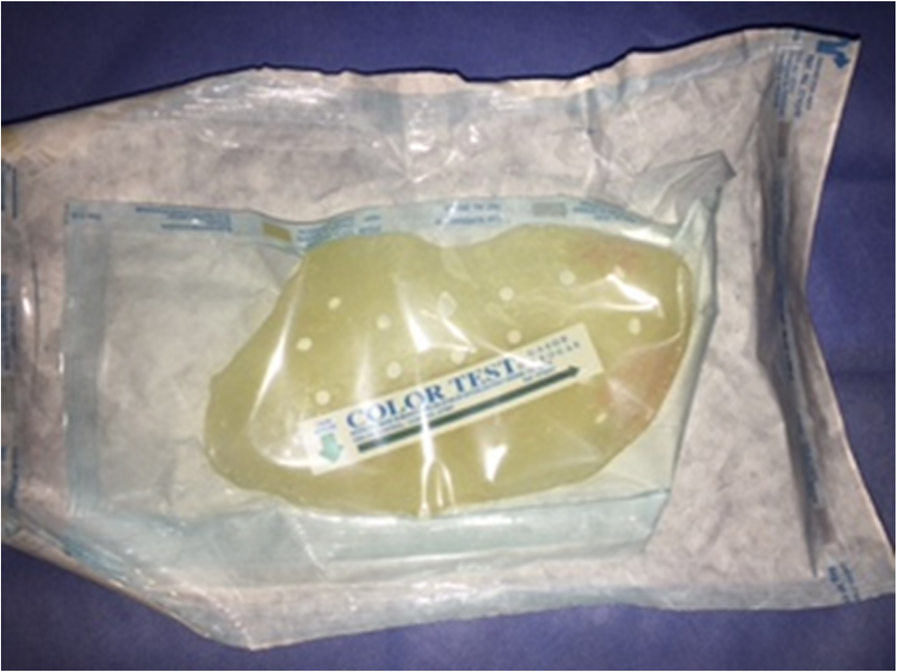
Ethylene oxide sterilized prosthesis

The prosthesis is placed in metal containers to obtain two plaster impressions (an internal and an external surface impression). A transparent PMMA OPTI-CRYL^R^ is poured into the space created by the internal plaster impression and pressed with the external mold. The casts are opened and the prosthesis is revealed after a 25-min polymerization time (Fig. 8). The prosthesis is then cut, perforated, and polished with a low-speed motor. Precise anatomical compliance to the model is verified and approved by the multi-disciplinary team (including the neurosurgeons), and the prosthesis is then placed on a mannequin for academic and illustrative purposes (Fig. 9). After obtaining the prosthesis in the laboratory, it is washed with normal saline solution and then submerged in a chlorine based antiseptic solution (Microdacyn 60 by Oculus lnnovative Sciences in Petaluma, CA.) for 15 min before taking it to the hospital where it is sterilized with ethylene oxide gas before its implantation (Fig. 10).

### Surgical technique

**Fig. 11:**
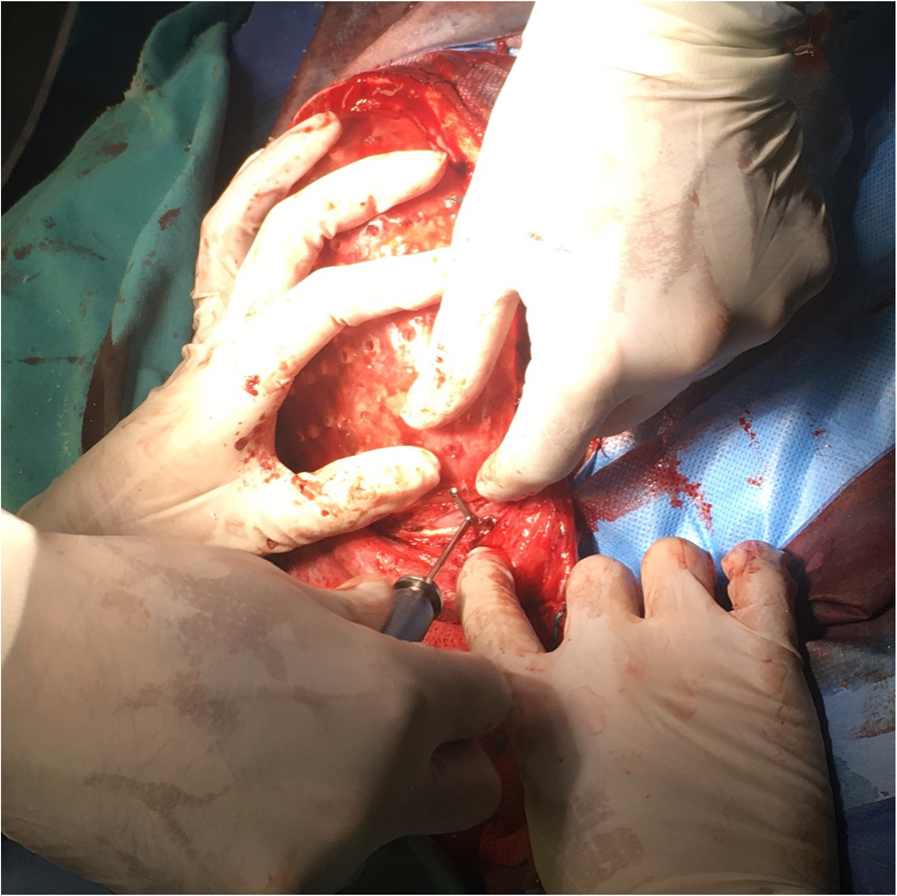
Placement and fixation with titanium plates

**Fig. 12:**
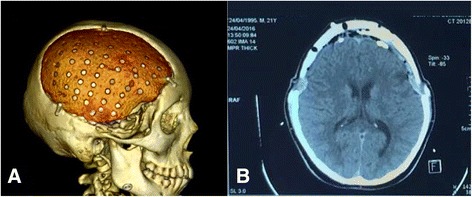
**A** Case Report 1 postoperative CT. Notice the coaptation of the prosthesis to the bone defect. **B** Case Report 2 postoperative CT. Notice the expansion of the frontal lobes on these axial plane images which were collapsed preoperatively

Cranioplasties were carried out 3 months after craniotomy in conjunction with the neurosurgeons. The prostheses were then placed over each of the defects to adjust them in vivo with minimum adjustments with the same low-speed electric motor, and fixed with 3 long titanium bridge plates, each held down with 2 screws (Fig. 11). The procedure ended without any unexpected events. The patients were hospitalized for 2 days and discharged after a single drain is removed from each patient to continue their care as outpatients (Fig. 12).

**Fig. 13:**
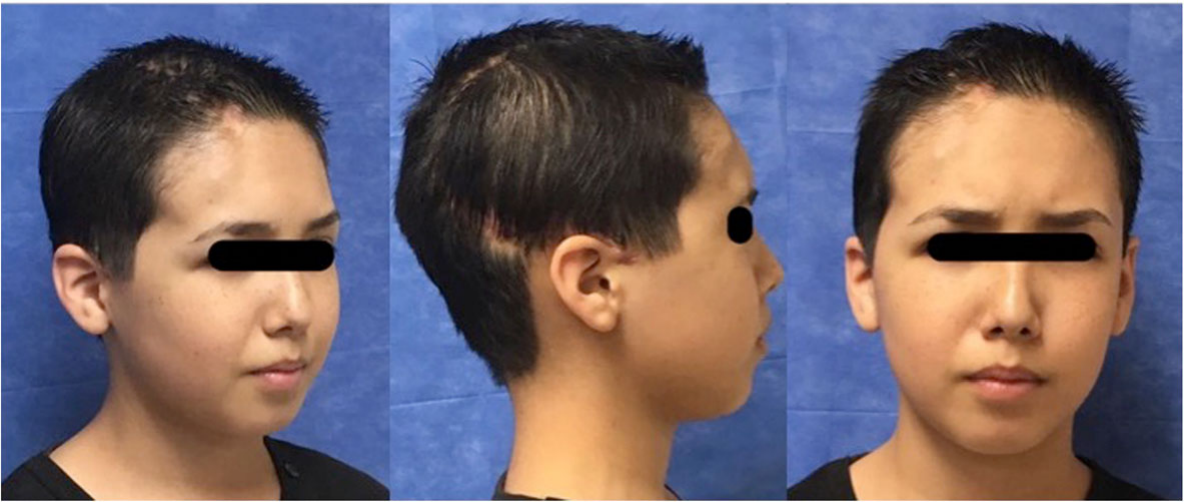
6-month postoperative follow-up on our patient in Case 1 shows a great cranial symmetry, and wound healing

The patients were evaluated every week for 6 months to register PMMA behaviour, biological sefety and any eventualities (Fig. 13).

## Results

Two patients, both with large cranial defects (> 100 cm^2^) underwent a cranioplasty with individually customized prefabricated molds generated from a 3D printed template. A description of some main characteristics of the cranial prostheses can be seen in Table 1.

**Table 1:** Description of cranial prostheses characteristics

Analysis	Case 1	Case 2
Intraoperative adjustment time	8 min	16 min
Precision to cranial defect	Exact	Exact
Intraoperative exothermic reaction	None	None
Cranial symmetry	Exact	Exact
Cost	Low	Low

The left-hand column specifies the parameters that were evaluated on each prosthesis. This type of cranial vault reconstruction requires little trans-operative adjustment time, since most adjustments are done pre-operatively, and the prosthesis adapts precisely to the defect (this was measured by tracing the prosthesis and its adjustments on to a millimeter paper), restoring cranial symmetry to perfection (a comparison of the intact side against the side with the defect) without the burden of an exothermic reaction on brain tissue, and minimal production cost

There were no adverse events such as prosthesis exposure, seroma, infections, nor any toxicity or encephalic inflammation since the cerebral tissue was not exposed to an exothermic reaction in the OR. After a 6 month post-operative follow-up, cranioplasty patients had an appropriate neurological progression defined by measuring their cognitive and motor skills.

## Discussion

Hand-manufacturing of a prosthetic implant can only be carried out if the original cranial bone fragment is available. Since the cases treated at our Hospital presented with fragmented cranial fractures, bone preservation was implausible.

PMMA prosthesis manufacturing by hand has been used since 1970 using various processes [1, 16–18], but these methods have been overtaken by computer assisted design and manufacturing techniques (CAD/CAM) which consist on using images of the cranial defect and manufacturing the PMMA prostheses with a 3D printer [19–25]. The CAD/CAM technique described by Caro-Osorio & Cols. [26] in 2013 is no longer an expensive method. The ideal implant material should have the following characteristics: it should be able to adjust to the cranial defect, it must achieve a full defect coverage, full biocompatibility, inertness, non-thermal conductor, radiolucent, non-magnetic, light, rigid, easily placed and low-cost [19, 27].

A titanium prosthesis [14] is more difficult to manufacture than one made from PMMA [28]. Even though, as Park and cols. [29] have commented, postoperative patients treated with a titanium implant have a satisfactory postoperative course evaluated with CTs, documenting an adequate implant fixation and cranial symmetry, the main obstacle with this technique is cost.

Preoperative prosthesis manufacturing is simpler, technically speaking, and has the added advantage of lowering surgical time, blood loss and infection rate, and improving the aesthetic result satisfaction when compared to intraoperative moulding [30].

PMMA preoperative implant manufacturing can be done either by hand or with CAD/CAM techniques. Manufacturing done by hand is cheaper and less time consuming than using a patient’s 3D-CT data to then print a 3D implant [21, 24]. Nevertheless, this last method has recently gained popularity because it does not require to be tried on the bone defect to produce excellent cosmetic results [30].

Pre-operative production (ie. prefabrication) of a cranioplasty prosthesis involves a computer-aided design system and direct computer-aided manufacturing to obtain the desired shape. This system uses standard 3D CT data.

It is done by locating the cranial defect margin on a skull surface image generated from a 3D head CT-scan. A right-to-left mirrored image or an average 3D skull surface template image is then fitted to the patient’s skull surface image. The area around the defect is cut out and stitched to the previously isolated defect margin. This defect-filling surface is then tapered and printed out in 3D. The 3D print implant model is then recasted in a biocompatible material [21].

The process differs from ours in that the delineation and the stabilization of the skull defect margin are not required to identify the thickness and the shape of the implant piece. We use the Boolean operation of extraction exclusively between the mirrored side and the side with the defect to obtain the implant piece with the shape and the thickness required.

No complications were observed in either of the cases reviewed in this article, but the population cannot be presumed to be of any statistical significance. Thien A. and cols. [31] reported in 2015 a 25% tendency in incidence towards titanium prosthesis exposition in contrast to a 12.5% incidence with PEEK. These authors also mentioned that infection rate in patients undergoing decompressive craniotomy was associated with previous cranioplasties [31]. It is evident after reviewing the literature that there is no perfect prosthetic material for cranioplasties free from complications.

The results observed in our cases match the reports from Akam M. and cols. [32] who concluded that polymethyl methacrylate is a low-cost, long-lasting material which can be used to reconstruct full thickness cranial defects.

## Conclusions

For years, prefabricated customized cranial prostheses using 3D printers have demonstrated their use and advantage when compared to other techniques. In the Hospital Civil de Guadalajara Fray Antonio Alcalde, a group comprised of researchers and surgeons work together to obtain low-cost customized implants since titanium and PEEK (polyetheretherketone) prosthesis are too expensive for the type of population we treat.

This study proves the efficacy of customized PMMA prefabricated 3D prosthesis. Cranial symmetry is achieved to perfection, little intraoperative time is needed for adjustments, coaptation is precise. Lastly, compared to PMMA prostheses produced intraoperatively, no exothermal reaction is generated in the operating room, nor are any monomers liberated through gas formation, polymerization heat is avoided and adjustment time is reduced to a minimum.

The main limiting factor in this method is the generation of the DICOM file from the 3D CT since we do not own a bone scanner and we rely entirely on the hospital’s CT scanner availability.

This method allows an encouraging functional, aesthetic, biologically safe and low-cost patient treatment, especially to a low-income patient population like the one that is treated in our facilities, who would not otherwise have the means to pay for their health needs.

## Abbreviations

3D: Three dimensional
CAD/CAM: Computer-aided design/computer-aided manufacturing
CT: Computed tomography
DICOM: Digital images and communications in medicine
PEEK: PolyEtherEtherKetone
PLA: Poly-lactic acid
PMMA: PolyMethyl methacrylate
STL: Stereolithography
